# Antipsychotics with similar association kinetics at dopamine D_2_ receptors differ in extrapyramidal side-effects

**DOI:** 10.1038/s41467-018-04489-x

**Published:** 2018-09-03

**Authors:** Hugo Zeberg, Kristoffer Sahlholm

**Affiliations:** Department of Neuroscience, Karolinska Institutet, Stockholm, SE-17177 Sweden

## Introduction

The recent publication in *Nature Communications* by Sykes et al.^1^ made the interesting observation that forward binding rate constants (*k*_on_) at the dopamine D_2_ receptor (D_2_R) differ widely between antipsychotics, and moreover, that *k*_on_ rather than *k*_off_ (dissociation rate constant) values correlate with their liabilities to produce extrapyramidal symptoms (EPS). A high *k*_on_ is postulated to cause a high degree of rebinding of the antipsychotic to D_2_R, conferring increased competition with endogenous dopamine, and thereby more EPS. As pointed out by the authors, their results are largely in agreement with our previous findings from a potassium channel (G protein-activated inward rectifier potassium; GIRK) activation assay^2^. However, there are some discrepancies between their work and ours which may have important consequences for the interpretation of the correlation between *k*_on_ and EPS, and which we would like to point out below.

**Fig. 1:**
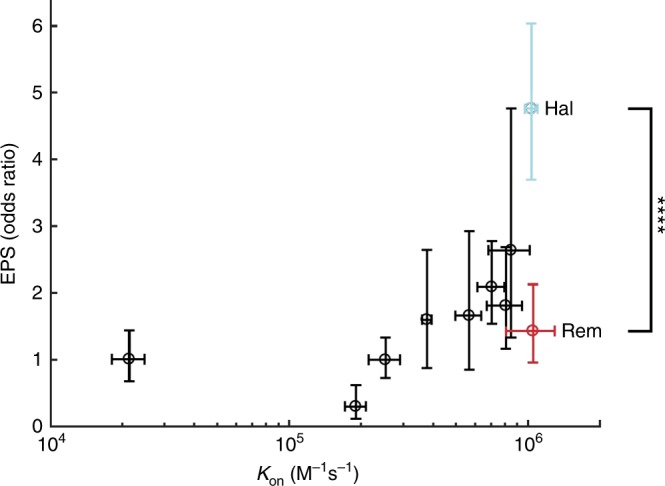
Correlation plot showing the relationship between *k*_on_ (from Sahlholm et al.^2^) and EPS odds ratio (from Leucht et al.^8^ and Lewander et al.^3^). The plotted antipsychotics are, from left to right: quetiapine, clozapine, olanzapine, amisulpride, asenapine, risperidone, paliperidone, chlorpromazine, haloperidol (Hal; turquoise), and remoxipride (Rem; red). ****p < 0.0001; *p* value calculated using Fisher’s exact test on the basis of data from Lewander et al.^3^ Vertical error bars represent credible intervals for the EPS odds ratios from the Bayesian hierarchical model by Leucht et al.^8^ (212 studies including 43049 patients), except for remoxipride, where the error bars represent a confidence interval calculated by extending the dataset of Leucht et al.^8^, by incorporating data from Lewander et al.^3^ (437 patients receiving haloperidol and 667 patients receiving remoxipride), as described in Methods. Horizontal error bars represent S.E.M. of the *k*_on_ estimates (based on data from 9 to 30 oocytes for each antipsychotic) and reflect the uncertainty of the linear regression of data from Sahlholm et al.^2^

Remoxipride, a clinically effective antipsychotic which was withdrawn due to the occurrence of aplastic anemia as a rare side-effect, has a very low rate of EPS compared to the typical antipsychotic, haloperidol^3^ (odds ratio for rigidity, 0.30; 95% CI, 0.22 to 0.41; *n* = 1104; *P* < 0.0001, Fisher’s exact test; Fig. 1). Importantly, unlike many other antipsychotics which target multiple types of monoamine receptors, both of these compounds preferentially bind to dopamine D_2_-like receptors^4^. Thus, the difference in EPS liability between remoxipride and haloperidol cannot easily be attributed to differential engagement of muscarinic or serotonergic receptors, which are thought to contribute to the low EPS potential of many of the newer antipsychotics, such as clozapine, quetiapine, and olanzapine^4–6^. This should make remoxipride and haloperidol particularly suitable for comparison based on their *k*_on_ values at D_2_R. Interestingly, in our hands, remoxipride has a high *k*_on_, similar to that of haloperidol^2^ (Fig. 1), and a very rapid *k*_off_, more than 5 times faster than that of clozapine. In the study by Sykes et al.^1^, however, remoxipride *k*_on_ is among the lowest in their dataset, thus fitting their *k*_on_ hypothesis, while they report a *k*_off_ for remoxipride which is similar to that of clozapine. It should be mentioned that remoxipride does not induce direct blockade of potassium currents up to a concentration of at least 300 µM, thus ruling out a potentially confounding effect of direct GIRK channel block on the measurement of D_2_R binding kinetics in our assay^7^.

Plotting our own *k*_on_ data against EPS odds ratios taken from Leucht et al.^8^ yields a similar positive correlation (Fig. 1) as that reported by Sykes et al.^1^ However, considering the kinetic properties of remoxipride found in our study, along with its low EPS liability, the correlation between *k*_on_ and said side-effects becomes less striking. We have inserted data for remoxipride vs. placebo (odds ratio, 1.43; 95% CI, 0.96 to 2.13; see Methods), which was not included in the data set presented by Leucht et al.^8^, based on the odds ratio for rigidity given above. When analyzing data for the 9 compounds which were included both in our study^2^ and that of Leucht et al.^8^, the correlation is strong (Spearman’s *R*_s_ = 0.93; *P* < 0.001), but is weakened by the inclusion of remoxipride (Spearman’s *R*_s_ = 0.70; *P* = 0.03).

The reasons for the discrepancies in remoxipride *k*_on_ and *k*_off_ between our study^2^ and that of Sykes et al.^1^ may relate to the differential kinetics of the probe ligands employed in the two studies–as pointed out in a previous publication by the same authors^9^, the kinetics of fast-dissociating ligands cannot by accurately measured by a much slower-dissociating probe ligand. It is thus relevant to note that the dissociation T_1/2_ of PPHT-red, the fluorescent ligand used to probe receptor-ligand interactions, is about 80 s (*k*_off_ = 0.52 min^−1^)^1^, whereas the kinetics of the dopamine response in our potassium channel assay is considerable faster, with a deactivation T_1/2_ of just below 3 s and an activation T_1/2_ below 1 s^10^. For comparison, the T_1/2_ which we measured for remoxipride was 3.4 s^2^, whereas Sykes et al.^1^ reported it as 0.36 min (21.6 s). Thus, estimates of the very rapid binding kinetics of remoxipride may have been distorted by the relatively slow kinetics of PPHT-red. In addition, the differences in kinetic parameters measured for remoxipride may also reflect the differences in receptor state–whereas our assay measures the functional, high-affinity state of D_2_R in living cells, the study by Sykes et al.^1^ used GppNHp to convert all D_2_R in their membrane preparation to the non-signaling, low-affinity state. We consider that drug interactions with the functional state of the receptor might be more relevant to the occurrence of side-effects. Whereas dopamine binding to D_2_R is known to be sensitive to the transmembrane voltage, such that the EC_50_ for dopamine-induced GIRK activation is about 4.2-fold higher at 0 mV compared to −80 mV^10^, we found the IC_50_ of remoxipride to inhibit the GIRK response to 100 nM dopamine to be 4.8-fold lower at 0 mV (IC_50_ 202.2 nM; pIC_50_ 6.69 ± 0.11; *n* = 5) compared to −80 mV (IC_50_ 963.5 nM; pIC_50_ 6.02 ± 0.09; *n* = 5). The IC_50_ value at −80 mV has been published previously^7^. This would be consistent with a competitive interaction between dopamine and remoxipride, where the affinity of remoxipride itself is not substantially altered by transmembrane voltage. Thus, a difference in remoxipride binding kinetics related to differential membrane potentials (the transmembrane voltage across isolated membranes, as used by Sykes et al.^1^, would be 0 mV, while the whole cells used in our study were clamped at a more physiological −80 mV^2^), would seem unlikely, although a reciprocal change in both *k*_on_ and *k*_off_ cannot be ruled out. Lastly, we note that thioridazine, which had been placed in the uncertain typical/atypical category^1^, has among the highest *k*_on_ values reported by Sykes et al.^1^, yet it has consistently been reported to have a favorable profile in terms of EPS^11^.

In summary, while we find the concept of rebinding, as discussed by Sykes et al.^1^, to be of great interest and of potential utility to the field of neuropharmacology, we believe that *k*_on_ is not the only factor governing EPS liability, even when considering D_2_R-preferring compounds. This is illustrated by comparing haloperidol and remoxipride, which despite having similar *k*_on_ at D_2_R in our hands, show very distinct EPS profiles in the clinic.

## Methods

### Electrophysiology data acquisition

The IC_50_ of remoxipride at −80 and 0 mV was determined using two-electrode voltage clamp in *Xenopus* oocytes expressing the human D_2_R together with RGS-4 and GIRK1/4 channels, measuring the GIRK current amplitude as a readout of D_2_R occupancy by dopamine^2, 7, 10^. For concentration-response data, oocytes were randomly clamped at either −80 or 0 mV and exposed first to 100 nM dopamine, to evoke GIRK activation. Thereafter, four increasing concentrations (10 nM–10 µM) of remoxipride were applied consecutively, in the continued presence of 100 nM dopamine, at 50-s intervals. The current amplitude at the end of each antagonist application interval was normalized to the control response to 100 nM dopamine, within every oocyte. *k*_on_ values were also obtained using the GIRK assay, and were taken from our previous work^2^. Oocyte harvesting from female *Xenopus laevis* toads was performed in accordance with the Guide for Care and Use of Laboratory Animals as adopted and promulgated by the United States National Institutes of Health. Prior approval for the procedure was granted by the Swedish National Board for Laboratory Animals and the local ethics committee; Stockholms Norra Djurförsöksetiska nämnd.

### Odds ratio calculation

The odds ratio for remoxipride vs. haloperidol was obtained from EPS (rigidity) incidences and number of patients reported by Lewander et al.^3^ The odds ratio for remoxipride vs. placebo was calculated as

1 $${\mathrm{OR}}\left( {R{\mathrm{|}}P} \right) = \frac{{R_{{\mathrm{EPS}} + }/R_{{\mathrm{EPS}} - }}}{{P_{{\mathrm{EPS}} + }/P_{{\mathrm{EPS}} - }}} =$$

$$\left( {\frac{{R_{{\mathrm{EPS}} + }/R_{{\mathrm{EPS}} - }}}{{H_{{\mathrm{EPS}} + }/H_{{\mathrm{EPS}} - }}}} \right) \times \left( {\frac{{H_{{\mathrm{EPS}} + }/H_{{\mathrm{EPS}} - }}}{{P_{{\mathrm{EPS}} + }/P_{{\mathrm{EPS}} - }}}} \right) = {\mathrm{OR}}\left( {R{\mathrm{|}}H} \right) \times {\mathrm{OR}}\left( {H{\mathrm{|}}P} \right)$$

where $R_{{\mathrm{EPS}} \pm }$, $H_{{\mathrm{EPS}} \pm }$, and $P_{{\mathrm{EPS}} \pm }$ denote number of patients treated with remoxipride, haloperidol, and placebo, with our with or without EPS, respectively. Given the relationship above (1), the standard error ${\mathrm{SE}}_{\log {\mathrm{OR}}\left( {R|P} \right)}$ propagates according to

2 $${\mathrm{SE}}_{\log {\mathrm{OR}}\left( {R|P} \right)} = \sqrt {{\mathrm{SE}}_{\log {\mathrm{OR}}\left( {R|H} \right)}^2 + {\mathrm{SE}}_{\log {\mathrm{OR}}\left( {H|P} \right)}^2}$$

which was used to calculate the confidence interval for remoxipride. This extension of the data set to include remoxipride did not use the Bayesian hierarchical model employed by Leucht et al.^8^ However, the odds ratio calculated here should provide a good estimate for comparison with the antipsychotics in the original analysis.

### Statistical analysis

The statistical significance of the differential proportions of patients experiencing EPS between haloperidol-treated and remoxipride-treated subjects (data from Lewander et al.^3^) was calculated using Fisher’s exact test.

### Data availability

All relevant data are available from the authors upon request.
